# Responses of a legume to inbreeding and the intensity of novel and familiar stresses

**DOI:** 10.1002/ece3.4831

**Published:** 2019-01-15

**Authors:** Finn Rehling, Diethart Matthies, Tobias Michael Sandner

**Affiliations:** ^1^ Department of Nature Conservation, Faculty of Biology Philipps‐University Marburg Marburg Germany; ^2^ Department of Ecology, Faculty of Biology Philipps‐University Marburg Marburg Germany

**Keywords:** biomass allocation, functional traits, inbreeding depression, purging, rhizobia

## Abstract

It is often assumed that the negative effects of inbreeding on fitness (inbreeding depression, ID) are particularly strong under stressful conditions. However, ID may be relatively mild under types of stress that plant populations have experienced for a long time, because environment‐specific deleterious alleles may already have been purged. We examined the performance of open‐ and self‐pollinated progeny of the short‐lived calcareous grassland plant *Anthyllis vulneraria *under three intensities of each of five types of stress. Drought, nutrient deficiency, and defoliation were chosen as stresses typical for the habitat of origin, while shade and waterlogging were expected to be novel, unfamiliar stresses for *A. vulneraria*. The stresses reduced plant biomass by up to 91%, and the responses of the plants were mostly in line with the functional equilibrium hypothesis. There was significant ID in biomass (*δ* = 0.17), leaf chlorophyll content, and the number of root nodules of the legume, but the magnitude of ID was independent of the stress treatments. In particular, there was no significant interaction between inbreeding and the intensity of any stress type, and ID was not higher under novel than under familiar stresses. In addition, phenotypic plasticity in biomass allocation, leaf functional traits and in root nodulation of the legume to the various stress treatments was not influenced by inbreeding. Our findings do not support the common hypothesis of stronger ID under stressful environments, not even if the stresses are novel to the plants.

## 1 INTRODUCTION

The mating of closely related individuals can severely affect offspring fitness, a phenomenon called inbreeding depression (ID; Charlesworth & Charlesworth, 1987; Darwin, 1876). Inbreeding increases homozygosity, and ID is mostly caused by the expression of recessive deleterious alleles that are masked by heterozygosity in crossed progeny (Charlesworth & Charlesworth, 1987). Estimating the strength of ID in natural populations is of interest for both conservation and evolutionary biology, because ID plays an important role in the extinction of populations (Gilpin & Soulé, 1986; Hedrick & Kalinowski, 2000) and in the evolution of mating systems (Charlesworth & Charlesworth, 2010).

The magnitude of ID often differs among environments (e.g., Cheptou & Donohue, 2011) and has sometimes been found to be particularly strong under stressful conditions (Armbruster & Reed, 2005; Dudash, 1990; Fox & Reed, 2011; Hauser & Loeschke, 1996). Selfed offspring may be less capable to resist abiotic and biotic stressors and less effective in utilizing resources than crossed offspring (Reed, Fox, Enders, & Kristensen, 2012). However, there are many exceptions to this general pattern (Armbruster & Reed, 2005; Pemberton, Ellis, Pilkington, & Bérénos, 2017), and different types of stress may have different effects on ID (Daehler, 1999; Sandner & Matthies, 2016; Walisch, Colling, Poncelet, & Matthies, 2012; Waller, Dole, & Bersch, 2008). ID can even be higher under more benign conditions when crossed and selfed plants suffer similarly from a stressful environment, while crossed plants are more capable of taking advantage of benign conditions (Cheptou & Donohue, 2011; Sandner & Matthies, 2016). For example, fertilization of plants may increase ID compared to more nutrient‐poor conditions (Daehler, 1999; Hayes, Winsor, & Stephenson, 2005; Kéry, Matthies, & Spillmann, 2000; Sandner & Matthies, 2016; Walisch et al., 2012), and in a hemiparasitic plant, ID was greater when grown with good hosts (Sandner & Matthies, 2017). However, it is not yet understood which types of stress potentially increase or reduce the magnitude of ID.

It has been proposed that ID should be relatively low under types of stress that a population has been exposed to for a long time (Cheptou & Donohue, 2011; Reed et al., 2012). This is expected because selection should eliminate alleles that have detrimental effects on fitness in such “familiar” stressful environments (Agrawal & Whitlock, 2010; Bijlsma, Bundgaard, & Putten, 1999; Pemberton et al., 2017). In contrast, ID should be higher under novel stresses, because alleles that are deleterious only under those conditions may not have been purged. Consistent with these expectations, populations of *Drosophila melanogaster *adapted to salt showed higher ID when reared on cadmium‐enriched than on salt‐enriched media, although not *vice versa* (Long, Rowe, & Agrawal, 2013), and ID in *Silene vulgaris *was lower under types of stress frequently occurring in the population of origin than under novel stresses (Sandner & Matthies, 2016). However, there are no studies that systematically compare the effects of increasing intensities of novel and familiar types of stress on ID in plants.

Effects of inbreeding on the ability of plants to phenotypically respond to environments might be a key factor explaining differences in ID among environments. However, few studies have investigated the effects of inbreeding on the expression of functional plant traits in response to stresses. In *Solanum carolinense*, inbreeding reduced physiological and defense responses to herbivory (Campbell, Halitschke, Thaler, & Kessler, 2014), and in *S. vulgaris *inbreeding reduced the plasticity of leaf area and chlorophyll content, particularly in response to shade (Sandner and Matthies 2018). In contrast, other studies did not find consistent effects of inbreeding on the response of plant functional traits to different environments (Murren & Dudash, 2012; O'Halloran & Carr, 2010; Schlichting & Levin, 1986). The importance of phenotypic plasticity in functional traits for environment‐dependent ID thus requires further research.

It has been suggested that ID may increase under some environments not because they are more stressful, but because they increase the phenotypic variation among plants and thus the opportunity for selection (Waller et al., 2008). For example, some environments can increase and others decrease size differences among plants (Weiner, 1985), which in turn may increase or decrease ID (Cheptou, Lepart, & Escarré, 2001; Sandner & Matthies, 2016; Schmitt & Ehrhardt, 1990). The effects of the environments on size differences and phenotypic variation should thus be tested as a null‐model in studies on environment‐dependent ID in plants (Sandner & Matthies, 2016; Waller et al., 2008).

To study the effects of different intensities of various types of stress on ID, we grew open‐ and self‐pollinated offspring of the calcareous grassland plant *Anthyllis vulneraria *under three levels of each of five types of stress. Three of the stresses (drought, nutrient deficiency, and defoliation) are common in the habitat of the species and were thus considered familiar. Two other stresses (shade and waterlogging) do not occur in the original habitat and were considered to be novel stresses for *A. vulneraria*. Although every plant is familiar with some degree of shading by competitors, an intensive and long‐lasting shade can be regarded as familiar only for specialized understorey plants. Similarly, abundant water may be regarded as the absence of drought, but permanent waterlogging is a stress that requires specific responses, as it leads to hypoxia in the root system and the accumulation of products of anaerobic metabolism by micro‐organisms (Jackson & Colmer, 2005). Waterlogging may thus be familiar for wetland species, but not for *A. vulneraria*. We addressed the following questions: (a) Does ID in *A. vulneraria *increase with the strength of novel stresses, but not with that of stresses typical for the habitat of *A. vulneraria*? (b) Does ID increase under conditions that increase the phenotypic variation or initial size differences among plants? (c) Does inbreeding affect functional traits involved in the stress response of *A. vulneraria*?

## 2 MATERIAL AND METHODS

### 2.1 Study species

*Anthyllis vulneraria* L. (Fabaceae) grows in dry calcareous grasslands throughout most of Europe (Hegi & Gams, 1975). Typical stresses in these habitats are drought, nutrient deficiency, and herbivory. *A. vulneraria *is a monocarpic legume that flowers after 1–5 years (Bastrenta, Lebreton, & Thompson, 1995; Davison et al., 2010; Sterk, Duijkeren, Hogervorst, & Verbeek, 1982). In some populations, *A. vulneraria* was found to reproduce predominantly by self‐pollination (Couderc & Gorenflot, 1978; Sterk et al., 1982). In contrast, other studies have found that autogamy in *A. vulneraria* can occur, but is mostly prevented by protandry or gynodioecy (Navarro, 1999) and that reproduction depends on cross‐pollination (Helsen, Jacquemyn, & Honnay, 2015; Kesselring, Hamann, Stöcklin, & Armbruster, 2013; Navarro, 1999; Van Glabeke, Coart, Honnay, & Roldán‐Ruiz, 2007). The most important pollinators are bees of the genus *Anthophora* (Navarro, 2000). The fruits of *A. vulneraria* contain one large seed which is dispersed by animals (Hegi & Gams, 1975; Honnay et al., 2006). The morphology of *A. vulneraria* is very variable, and the species has been split into a large number of subspecies (Hegi & Gams, 1975; Puidet, Liira, Paal, Partel, & Pihu, 2005; Rola, 2012). However, molecular studies have questioned the validity of these subspecies (Köster, Bitocchi, Papa, & Pihu, 2008; Nanni, Ferradini, Taffetani, & Papa, 2004). *A. vulneraria *usually forms mutualistic symbioses with nitrogen‐fixing, determinate *Mesorhizobium *bacteria (Ampomah & Huss‐Danell, 2011; Ampomah et al., 2012; De Meyer, Hoorde, Vekeman, Braeckman, & Willems, 2011).

### 2.2 Pollination and stress experiment

In July 2011, seeds from 12 plants of *A. vulneraria* were collected in a meadow near Grän, Tirol, Austria (1,340 m a.s.l.). The plants grew at least 2 m apart from each other. In May 2012, we grew 1–3 offspring from each mother plant individually in a greenhouse and later in the Botanical Garden of the University of Marburg. In summer 2013, several inflorescences in the bud stage were covered with fine mesh cloth (1‐mm mesh size) to exclude pollinators. When the flowers opened, they were pollinated with pollen from other flowers of the same plant (self‐pollination treatment). Another set of inflorescences was not covered and freely accessible to pollinators (open‐pollination treatment). Some plants did not flower in the second year of growth and seeds from both self‐ and open‐pollination could be obtained from six of the original lineages. The ripe seeds were collected, and on average 22 seeds of each lineage and pollination type were weighed individually.

Seeds were scarified by cutting the seed coat with a scalpel and then placed on moist filter paper in Petri dishes at room temperature. The majority of seeds germinated within 3 days. In November 2013, seedlings were planted into 0.5 L pots filled with sand in a greenhouse. The seedlings were grown at a 20/10°C (day/night) temperature regime and received 12‐hr additional light from high‐pressure sodium lamps (Son T Agro 400 W). They were fertilized (125 mg per pot, N:P:K 14:7:14, “Hakaphos Gartenprofi”; Compo, Wien, Austria), and received water until saturation every second day.

After 15 days, seedlings from open‐ and self‐pollinations were subjected to five stress types at two levels (intermediate and high) or to a control treatment which represented a third level (low stress) for each stress type. This resulted in 30 treatment combinations (2 pollination types × 5 stress types × 3 stress levels) for which we used 10 replicates per combination. Only in the five control treatments, we used a total of 45 open‐pollinated and 49 self‐pollinated plants (instead of 50) due to a lack of seedlings. This resulted in a total of 294 plants. The treatment combinations were assigned to the plant lineages as equally as possible.

Plants in the control treatments were regularly watered, fertilized once a week (125 mg per pot) and received 12 hr of additional light per day. When plants were subjected to a stress treatment, the conditions were the same, except for the particular stress factor. In the drought treatments, pots were weighed every 3 days and watered until 13% (intermediate stress) or 7.5% (high stress) soil water content was reached. In the waterlogging treatments, the water level was kept at 4.5 cm (intermediate stress) or 1.5 cm (high stress) below the soil surface. In the nutrient deficiency treatments, 2 L of tap water was flushed through the sand before seedlings were planted into the sand to remove most of the nutrients. Plants then received weekly a diluted fertilizer solution containing only 1/8 of the amount of nutrients given to the control plants (intermediate stress), or only pure water (high stress). In the shade treatments, plants were covered with layers of neutral shading cloth and received only 32% (intermediate stress) or 19% (high stress) of the irradiation of the control plants. Finally, in the defoliation treatment all leaves were cut back by 75% (intermediate stress) or 100% (high stress) after 30 days of growth. These treatment levels were based on the results of a pilot study, in which *A. vulneraria *was grown under four levels of these stress types. The high level of each stress in the current experiment was chosen to strongly reduce biomass without killing the plants. The position of the plants in the greenhouse was re‐randomized once a week.

As an estimate of initial plant size, the number of leaves of each plant was counted and the length and width of each leaf measured before the stress treatments started (15 d). Initial leaf area per plant was calculated as the sum of the products of length and width of the leaves of a plant. Leaves of the young plants had only one leaflet. After 90 days, leaf chlorophyll content was measured with a chlorophyll meter (SPAD‐502; Minolta). Two days later, all plants were harvested. The number of leaves was counted, and the length of the longest petiole was measured. The leaves were cut at their base, weighed to obtain fresh mass and scanned to calculate total leaf area per plant. The stem was cut at ground level. The roots were carefully washed free of soil. For a subset of 215 individuals (*n* = 20–23 per pollination by stress type combination), the number of nodules was counted and the diameter of the largest nodule was measured with calipers. Roots, stems, and leaves were separately dried at 80°C for 4 days and weighed.

Specific leaf area (SLA) was calculated as the ratio between leaf area and leaf dry mass, and the dry matter content of leaves (LDMC) as dry leaf mass divided by fresh leaf mass. The chlorophyll content per area was calculated from the SPAD‐measurements (a) as 0.000552 + 0.000404 × a + 0.0000125 × a^2^ (Richardson, Duigan, & Berlyn, 2002) and multiplied by SLA to obtain chlorophyll content per dry mass. Root, stem, and leaf mass fractions were calculated as the proportion of total biomass allocated to roots, stems, and leaves, respectively. Nodule density was calculated as the number of nodules divided by root mass.

### 2.3 Data analysis

Inbreeding depression (*δ*) in a trait (*W*) was calculated as the relative difference between open‐pollinated progeny and selfed progeny using the formula *δ* = 1 − (*W*_selfed_/*W*_open‐pollinated_). Analyses of variance (ANOVA) were used to investigate the effect of pollination type on seed mass. Three‐way ANOVAs were used to analyze the effects of pollination type, stress type, and stress level on plant biomass. We used log biomass as the response variable to test for environment‐dependent ID, as ID describes the proportional reduction of fitness due to inbreeding. Constant ID in all treatments then corresponds to a constant effect of pollination type across stress treatments on log‐transformed biomass (Cheptou & Donohue, 2011). In contrast, differences in *δ* among environments would be indicated by a significant stress × pollination effect on log biomass. When plant lineage was included in the ANOVAs, all results remained qualitatively the same, and this factor was thus excluded from the analyses to simplify the models. To compare the effects of the diverse stresses, we calculated the stress intensity of a treatment as 1 − (*W*_stress_/*W*_control_), where *W*_stress_ is the geometric mean biomass of offspring from open‐pollination subjected to a specific stress treatment, and *W*_control_ the geometric mean biomass of offspring from open‐pollination grown in the respective control treatment (Fox & Reed, 2011; Sandner & Matthies, 2016). This measure describes the mean negative impact of a stress treatment on total biomass when no inbreeding occurs. In contrast to the factor “stress level” (*df* = 2, Table 1), “stress intensity” is a continuous variable (*df* = 1). Stress intensity was included in the ANOVA as a linear contrast explaining a part of the stress treatment effect. A significant interaction between the effects of pollination type and stress intensity on log biomass would indicate that ID changes with stress intensity. A significant three‐way interaction (pollination type × stress type × intensity) would indicate that the effects of stress intensity on ID differed depending on stress type.

**Table 1:** Analysis of variance of the effects of open‐versus self‐pollination, five types of stress and stress level (control, intermediate, or high) on the biomass of *Anthyllis vulneraria*

Source	Biomass	
	*df*	*F*
Stress type	4	43.14***
Novel versus familiar	1	132.91***
Rest	3	13.21***
Stress level	2	250.89***
Stress type × level	8	13.79***
Novel × level	2	32.95***
Rest × level	6	7.40***
Open versus Self‐pollination	1	14.69***
Pollination × Stress type	4	0.33
Pollination × novel	1	0.76
Pollination × rest	3	0.19
Pollination × Stress level	2	0.11
Pollination × Stress type × level	8	0.47
Pollination × novel × level	2	0.03
Pollination × rest × level	6	0.62
Residual	263	

Note

Stress type was split into the contrasts novel (waterlogging, shade) versus familiar types of stress (drought, nutrient deficiency, defoliation), and the remaining effect of stress type.

^***^ *p* < 0.001.

To test whether the effect of novel types of stress on ID differs from that of familiar stresses to which *A. vulneraria *is assumed to be adapted (Question 1), the overall effect of stress type was partitioned into a contrast of novel versus familiar types of stress, and the remaining effect of stress type. A significant three‐way interaction pollination type × novel versus familiar × intensity would indicate that the effects of stress intensity on ID differed between novel and familiar stresses.

To test whether changes in ID are due to differences among environments in the amount of phenotypic variation (Question 2), we related ID to the opportunity for selection (Waller et al., 2008). The opportunity for selection (CV^2^, Crow, 1958) in total dry mass was calculated as variance × mean^2^ separately for selfed and open‐pollinated plants of each stress type by stress level combination and then averaged across the pollination types. The ID and CV^2^‐values of the five control treatments were averaged, and the mean values per treatment (*n* = 11) were used as replicates.

We also tested whether differences in ID among environments may be due to the effects of the environment on size differences among plants. To obtain a measure for size differences among individuals, offspring of open‐pollinated plants was equally divided into large and small individuals based on their initial leaf area after 15 days of growth (prior to the stress application). Analogously to the coefficient of ID, a coefficient of size depression was calculated as 1 − (*W_small_/W_large_*) for each stress type by level combination (Sandner & Matthies, 2016). We averaged initial leaf area and total dry mass of open‐pollinated individuals at harvest for the five control treatments. By comparing only open‐pollinated plants differing in initial size, the coefficient of size depression illustrates how strong ID would differ among environments if selfed and crossed plants differed only in size and not in their stress response (Sandner & Matthies, 2016). ANOVAs were used to study the effect of stress type on CV^2^ and size depression. Linear regressions were used to analyze if stress intensity influences CV^2^ and size depression. Furthermore, CV^2^, size depression, and ID were related to each other.

We used three‐way ANOVAs to analyze the effects of pollination type, stress type, and stress level on functional traits of the offspring, for example, SLA, biomass allocation, and nodule density (Question 3). Effects of inbreeding on phenotypic plasticity in a trait would be indicated by a significant pollination type by stress interaction. If necessary, data were log‐transformed to ensure homoscedasticity and normally distributed residuals. To analyze the effects of pollination type, stress type, and stress level on the probability of the plants to form nodules, we used a generalized linear model with a logit link and binomial errors (analysis of deviance, Quinn & Keough, 2002). All statistical analyses were conducted with IBM 20.0 SPSS statistics (SPSS, Chicago, IL, USA).

## 3 RESULTS

### 3.1 Effects of inbreeding and stress on fitness‐related traits

The mass of seeds from selfed flowers was 11.7% lower than that of seeds from open‐pollinated flowers (*F*_1,276_ = 24.98, *p* < 0.001), and after 15 days of growth, the leaf area of inbred seedlings was 18.4% smaller than that of seedlings from open‐pollinations (*F*_1,291_ = 23.74, *p* < 0.001). At harvest, the biomass of offspring from self‐pollinations was on average 16.6% lower than that of offspring from open‐pollinated plants (1.33 vs. 1.12 g; Table 1). When leaf area after 15 days was included as a covariable in the analysis, the effect of inbreeding on biomass was less pronounced (*F*_1,262_ = 3.45, *p* = 0.06). Inbreeding also affected other fitness‐related traits of *A. vulneraria* (Table 2), it reduced the number of leaves by 7.8% (30.1 vs. 27.8), and total leaf area by 13.8% (102.8 vs. 88.6 cm^2^). When biomass at harvest was included as a covariable in the analysis, it explained a large proportion of the variation in those two traits, and inbreeding no longer influenced total leaf area (*F*_1,262_ = 0.73, *p* = 0.39) and the number of leaves (*F*_1, 62_ = 0.06, *p* = 0.81; see Supporting Information Table S1 in Appendix S1).

**Table 2:** Analyses of variance of the effects of open‐versus self‐pollination, stress type, and stress level (control, intermediate, or high) on fitness‐related traits (TLA, log‐total leaf area; NL, number of leaves), on allocation (LMF, leaf mass fraction; SMF, stem mass fraction; RMF, root mass fraction) and other functional plant traits (LDMC, leaf dry matter content; SLA, log‐specific leaf area; Pet, log‐petiole length; Chl, leaf chlorophyll content) of *Anthyllis vulneraria*

Source	*df*	Fitness traits		Functional plant traits						
		TLA	NL	LMF	SMF	RMF	LDMC	SLA	Pet	Chl
		*F*	*F*	*F*	*F*	*F*	*F*	*F*	*F*	*F*
Stress type	4	17.97***	17.29***	91.65***	42.31***	91.21***	65.88***	151.67***	14.27***	72.35***
Stress level	2	238.78***	193.20***	15.35***	34.33***	2.55	39.71***	7.28***	67.48***	44.23***
Stress type × level	8	7.88***	8.38***	28.93***	18.97***	30.41***	24.38***	57.54***	6.64***	24.68***
Open versus Self‐Pollination	1	11.31***	6.22*	0.32	7.68**	2.28	0.55	0.38	1.71	11.31***
Pollination × Stress type	4	0.39	0.81	0.49	0.52	0.33	0.26	0.09	0.37	0.63
Pollination × Stress level	2	0.01	1.07	0.04	0.27	0.13	0.05	0.71	0.02	0.20
Pollination × Stress type × level	8	0.62	0.38	0.43	1.75	1.02	1.18	1.14	1.33	0.74
Residual	263									

^*^ *p* < 0.05; ^**^ *p* < 0.01; ^***^ *p* < 0.001.

**Figure 1:**
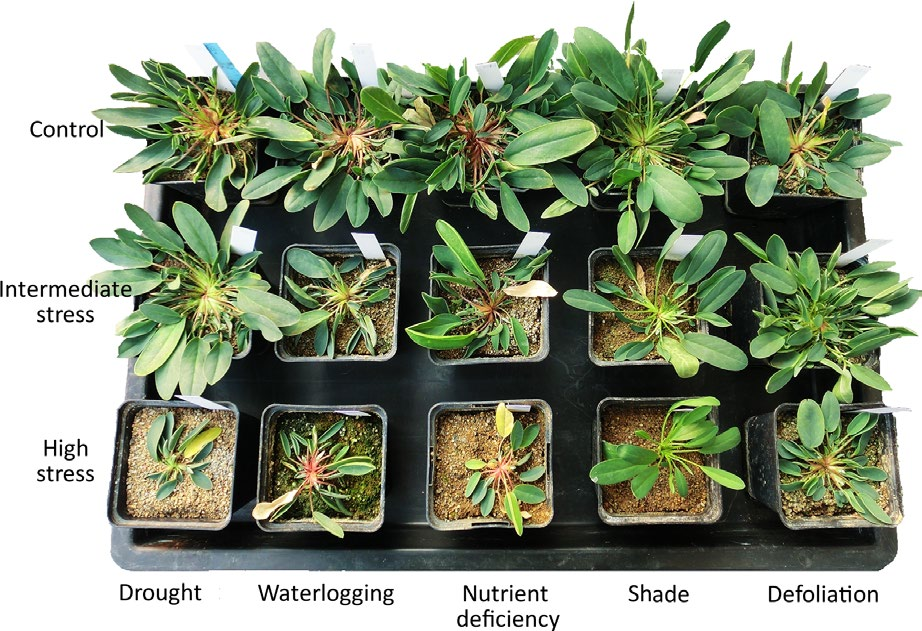
Individuals of *Anthyllis vulneraria* subjected to different types of stress (drought, waterlogging, nutrient deficiency, shade, and defoliation) at three levels (no stress = control, intermediate, high)

**Figure 2:**
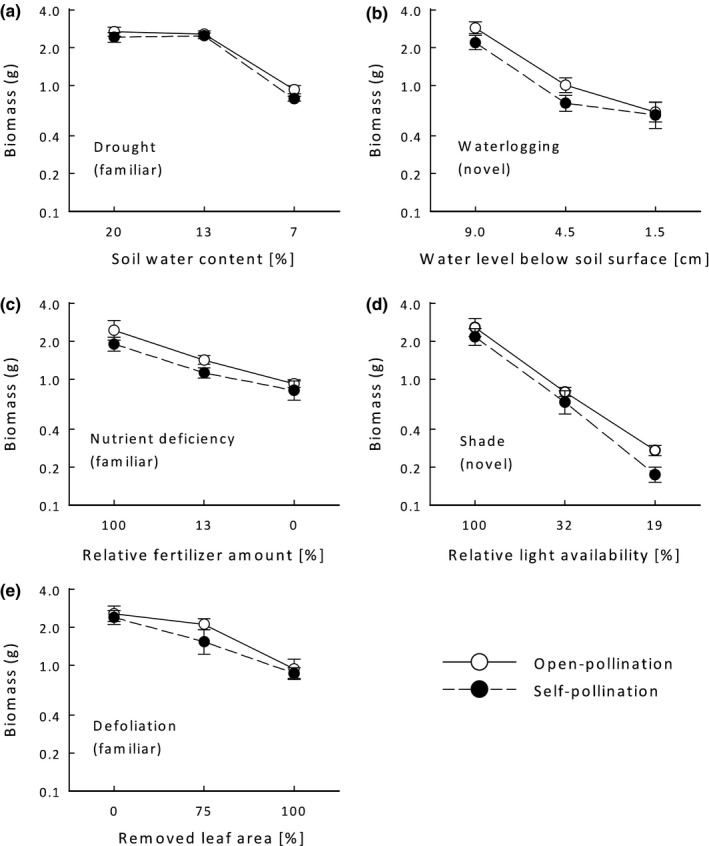
The effect of the intensity of five types of stress on the biomass of offspring from (○) open‐ and (●) self‐pollinations in *Anthyllis vulneraria*. Drought (a), nutrient deficiency (c), and defoliation (e) were expected to be types of stress that are familiar to *A. vulneraria*; waterlogging (b) and shade (d) were considered to be novel types of stress. Means ± 1 *SE*

In addition to inbreeding, the experimental stress treatments strongly influenced the growth of *A. vulneraria* (Figure 1). For each stress type, stronger stress levels reduced biomass more strongly than intermediate levels. However, the strength of the negative effect of increasing levels of stress on plant biomass depended on stress type (Table 1, Figure 2). The intermediate drought treatment had the smallest effect on plant biomass (−0.7%), while strong shade as the most adverse treatment reduced biomass by 90.8% compared to the control treatment. Similarly, stress affected the size‐related traits total leaf area and number of leaves (Table 2). Although stress intensities (measured as the negative effects of treatments on biomass) were high, only one of 294 plants died during the experiment (under strong nutrient deficiency).

Although both inbreeding and stress reduced plant biomass, neither the effects of stress type nor of stress level interacted with those of pollination type (Table 1). To make the levels of stress comparable across the different types of stress, in an additional analysis we replaced the stress levels of each treatment by their intensity (measured as the average negative effect of a specific treatment on biomass). ID did also not change with stress intensity (no interaction between pollination type and stress intensity; *F*_1,263_ = 0.015, *p* = 0.90). To test whether the effects of presumably novel types of stress to which *A. vulneraria* is not adapted differed from those of stresses that occur frequently in the habitats of the plant, we partitioned the effects of stress type into those of novel versus familiar stresses. The novel stresses affected the biomass of *A. vulneraria *more strongly than the familiar stresses, but ID did not depend on the novelty of a stress (Table 1). Moreover, the effect of stress intensity on ID did not differ between the two forms of stress (*F*_1,263_ = 0.062, *p* = 0.80).

We also investigated if the magnitude of ID depended on the effects of a stress treatment (*n* = 11) on phenotypic variation (CV^2^) or on size differences between initially large and small plants. Mean CV^2^ per treatment tended to differ among stress types (*F*_5,5 = _3.91, *p* = 0.08). Phenotypic variation in biomass was highest under waterlogging (CV^2^ = 0.28) and lowest under drought (CV^2^ = 0.04). However, CV^2^ was not significantly related to stress intensity (*r* = 0.23, *p* = 0.50), and ID was not related to mean CV^2^ per treatment (*r* = 0.01, *p* = 0.98). Size differences between large and small plants in leaf area decreased during the experiment. The leaf area of the 50% small plants was 38.6% smaller than that of the 50% large plants after 15 days, but only 17.4% smaller at harvest. The coefficient of size depression in biomass at harvest was not influenced by the type of stress (*F*_5,5_ = 0.41, *p* = 0.82) or stress intensity (*r* = 0.04, *p* = 0.90). In addition, size depression did not explain much variation in either CV^2^ (*r* = 0.13, *p* = 0.70) or ID (*r* = −0.39, *p* = 0.24).

### 3.2 Effects of stress treatments and inbreeding on functional traits of *A. vulneraria*

**Figure 3:**
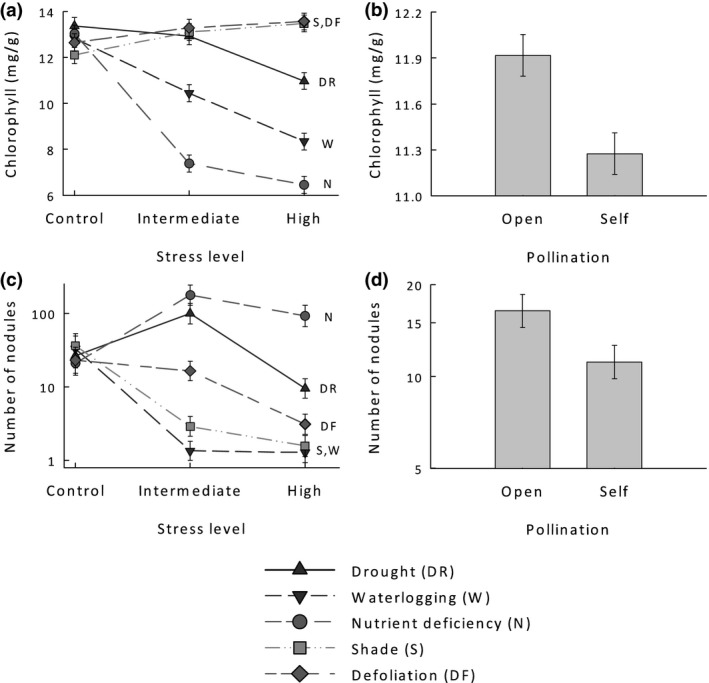
The effect of three levels (control, intermediate, and high) of five types of stress (drought, nutrient deficiency, defoliation, shade, and waterlogging) on (a) leaf chlorophyll content, and (c) the number of nodules of *Anthyllis vulneraria*. (b, d) The effect of inbreeding (open‐ vs. self‐pollination) on (b) leaf chlorophyll content, and (d) the number of nodules of *A. vulneraria*. Note the log‐scale for number of nodules. Means ± 1 *SE*.

All investigated plant traits were strongly influenced by the interaction of stress type and level (Table 2). In line with the functional equilibrium hypothesis, individuals of *A. vulneraria* changed their biomass allocation when subjected to different stress types. Under drought and nutrient deficiency, the root mass fraction of plants was increased compared to the control group, while plants subjected to defoliation, shade, and waterlogging invested more biomass into their above‐ground parts (see Supporting Information Figure S1 in Appendix S1). Compared to the control treatments, the leaf chlorophyll content was reduced under most stress treatments, but increased under shade and after defoliation (Figure 3a). The length of the longest petiole was reduced under all stress types other than shade, even though plant biomass was much smaller under shade than in the control (see Supporting Information Figure S2 in Appendix S1). Specific leaf area and leaf dry matter content (LDMC) were negatively correlated (*r* = 0.81, *p* < 0.001, *n* = 291). While SLA was increased under shade and defoliation but reduced in response to waterlogging, drought, and nutrient deficiency, LDMC was decreased under shade and defoliation and increased in the other treatments (see Supporting Information Figure S2 in Appendix S1).

Inbreeding affected some functional traits involved in the stress response of *A. vulneraria*. Leaf and root mass fraction did not differ between open‐ and self‐pollinated individuals (Table 2), but the stem mass fraction of self‐pollinated plants was higher than that of open‐pollinated plants (11.2% vs. 10.5%). After adjusting for the biomass of the plants, stem mass fraction was still influenced by inbreeding (see Supporting Information Table S1 in Appendix S1). Among leaf traits, only chlorophyll content was affected by inbreeding (Table 2). Leaves of self‐pollinated plants contained 5.4% less chlorophyll per leaf mass than those of open‐pollinated plants (Figure 3b). This effect remained significant after adjusting for differences in biomass (see Supporting Information Table S1 in Appendix S1). Inbreeding did not influence LDMC, the length of the longest petiole, or SLA (Table 2). Inbreeding did also not affect phenotypic plasticity of any of the traits, as there was no significant interaction between pollination type, stress type, and stress level for any of the functional plant traits studied (Table 2).

### 3.3 Effects of stress treatments and inbreeding on the nodulation of the legume

Nodule production by the roots varied strongly among individuals of the legume *A. vulneraria*. Under control conditions, 10.3% of the individuals did not form any nodules, whereas the other plants produced up to 665 nodules (geom. mean = 28.1). Nevertheless, there were strong effects of stress type and level on functional nodule traits (Table 3). Most of the nodule traits were positively influenced by nutrient deficiency and intermediate drought, but negatively affected by defoliation and strong drought, and especially by shade and waterlogging (Figure 3d, see Supporting Information Figure S2 in Appendix S1). Subjected to waterlogging, only 12.5% of plants were capable of forming nodules, and nodule number was lowest (Figure 3c).

**Table 3:** Analyses of variance of the effects of open‐versus self‐pollination, stress type, and stress level (control, intermediate, or high) on functional nodule traits (probability of nodule presence; log‐number of nodules, log‐nodule density; and the size of the largest nodule) of *Anthyllis vulneraria*

Source	*df*	Functional nodule traits				
		Prob. of presence	Number	Density	Size	
		Quasi‐*F*	*F*	*F*	*df*	*F*
Stress type	4	46.33***	49.78***	46.17***	4	21.26***
Stress level	2	3.29**	29.20***	9.94**	2	4.48*
Stress type × level	8	14.65***	13.03***	10.44***	8	7.56***
Open versus Self‐Pollination	1	0.01	4.03*	0.77	1	9.48**
Pollination × Stress type	4	1.24	2.07	1.83	4	1.28
Pollination × Stress level	2	0.03	0.67	0.76	2	0.79
Pollination × Stress type × level	8	0.22	0.24	0.35	8	0.48
Residual	185				132	

^*^ *p* < 0.05; ^**^ *p* < 0.01; ^***^ *p* < 0.001.

Inbreeding did influence the nodulation of the roots of *A. vulneraria *(Table 3). Although the probability that rhizobia inoculated the roots of *A. vulneraria *was not affected by inbreeding, inbreeding resulted in a strong reduction of the number of nodules per plant (−37.9%, Figure 3d) and reduced the size of the largest nodule by 15.9% (1.9 vs. 1.6 mm). However, inbreeding did not influence nodule density (Table 3), and the effect of inbreeding on maximum nodule size was reduced if adjusted for the number of nodules per plant (*F*_1,131_ = 3.89 *p* = 0.07). Inbreeding did also not influence the effect of the various stress treatments on root nodulation.

## 4 DISCUSSION

### 4.1 Environmental effects on inbreeding depression

Both inbreeding and environmental stresses reduced the biomass of *A. vulneraria*, but the magnitude of ID was not influenced by the type or level of stress. This is in contrast to the results of studies that found a higher sensitivity of selfed individuals to stressful environments (e.g., Dudash, 1990; references in Armbruster & Reed, 2005). In their meta‐analysis, Fox and Reed (2011) found a strong, positive correlation between ID and stress intensity and suggested that exceptions from this pattern may have been due to mild stresses which did not reduce total fitness by more than 25%. However, the higher levels of our stress types reduced total biomass of *A. vulneraria *on average by 72% and thus have to be considered as severe. The magnitude of ID observed (17%) is similar to the average ID in biomass and reproduction found for self‐compatible species (Husband & Schemske, 1996). Total ID will be higher, because it includes effects on seed set, germination, and flowering which we did not study. In addition, we may have underestimated the magnitude of ID by comparing self‐pollination with open‐pollination, which may to some degree include pollen transfer from flowers of the same plant (geitonogamy, De Jong, Waser, & Klinkhamer, 1993). However, open‐pollination rather than pure outcrossing is the normal case in natural populations, and the fact that we found ID in most studied traits suggests the expression of deleterious alleles. This justifies the expectation that if a general relationship between ID and stress intensity exists, ID should also increase in *A. vulneraria* in response to our stress treatments, which was not the case. Our findings are in line with those of other recent studies that investigated ID in individual species under several stresses of different intensity using *Drosophila melanogaster* (Yun & Agrawal, 2014), *S. vulgaris* (Sandner & Matthies, 2016) and *Rhinanthus alectorolophus *(Sandner & Matthies, 2017). There is thus little support for a linear increase of ID with stress intensity irrespective of the type of stress.

Different types of stress require different physiological responses, and it is thus possible that ID increases only with the intensity of some types of stress, but not others. It has been proposed that novel types of stress may increase ID, since recessive deleterious alleles which are expressed only under these environments have not been selected against. In contrast, familiar types of stress may not increase ID, because recessive deleterious alleles expressed only under these environments may already have been purged (Agrawal & Whitlock, 2010; Bijlsma et al., 1999; Cheptou & Donohue, 2011; Pemberton et al., 2017; Reed et al., 2012). *A. vulneraria *is a species of unshaded dry habitats that are never waterlogged. Therefore, purging of deleterious alleles expressed only under shade and waterlogging is not to be expected. However, the effects of these novel stresses on ID were not stronger than those of nutrient deficiency, drought, and defoliation, which represent familiar stresses to which *A. vulneraria *is presumably adapted. This suggests that either purging has not been efficient under familiar conditions, or purging has also reduced the genetic load under conditions we regarded as novel, or simply that in the studied lineages, no conditionally deleterious alleles were present that could have been purged only under some conditions.

Although there is experimental evidence that repeated generations of inbreeding can reduce the amount of subsequent ID by purging (Crnokrak & Barrett, 2002; Swindell & Bouzat, 2006), the efficiency of purging in wild populations is usually low (Byers & Waller, 1999; Keller & Waller, 2002; Leberg & Firmin, 2008). Simulations show that under constant conditions purging can be effective for strongly deleterious alleles. In contrast, the purging of mildly deleterious alleles is effective only at intermediate or large population sizes, depending on the intensity of inbreeding and the recessiveness and selective disadvantage of the involved alleles (Glémin, 2003). When conditionally deleterious alleles are involved, purging can also be less efficient when environmental conditions vary among years (Bijlsma et al., 1999, but see Porcher et al., 2009). In addition, there are two different explanations why purging may have also reduced ID expressed under stresses which we considered to be novel for the species. Firstly, our a priori classification of stresses may have been incorrect and the experience of low levels of the stresses we regarded as novel may already have led to purging. For example, deleterious alleles that negatively affect plant performance under strong shade may already have been purged under low levels of shade by competitors. Secondly, the applied types of stress may also have been too novel for *A. vulneraria*. When a stress is never encountered by a species, this species may not have genes specific for the response to this stress and deleterious alleles cannot accumulate. In this case, the response of *A. vulneraria* to shade and waterlogging may have consisted of general stress responses instead of specialized mechanisms, and purging of deleterious alleles may already have taken place under other types of stress. And finally, it is possible that the stresses were indeed novel to the species, but by chance no deleterious alleles expressed only under shade or waterlogging were present. The results of other studies do not consistently support the expectation of higher ID under novel compared to familiar conditions. Although in *S. vulgaris* ID was relatively low under familiar stresses, ID was not higher under the novel copper stress than under control conditions (Sandner & Matthies, 2016). Similarly, in a selection experiment with *Drosophila*, Long et al. (2013) found higher ID under the novel than under the familiar stress only for one selection environment (cadmium‐enriched), but not another (salt‐enriched). This suggests that novel stresses may not generally result in higher ID.

It has been suggested that differences in the magnitude of ID among environments may not be a consequence of stress itself, but of effects of the environments on phenotypic variation (CV^2^, Waller et al., 2008). For example, when inbred plants compete with their outbred relatives, they suffer not only from ID, but also from stronger competition by the larger outbred plants, which increases ID, a concept termed “dominance and suppression” (Schmitt & Ehrhardt, 1990; Yun & Agrawal, 2014). Similarly, any environment that reduces size differences between small and large individuals might reduce ID (Sandner & Matthies, 2016). However, in the current study, we found no support for either hypothesis. In *A. vulneraria*, initial size differences between large and small plants were reduced during the experiment. This may indicate that pot size and equal amounts of fertilizer for all plants limited the growth of large plants more strongly than that of small plants which may be an additional explanation for the generally low levels of ID in our experiment. However, stress type and stress intensity did not significantly influence size differences among plants. The phenotypic variation (CV^2^) in biomass was highest under waterlogging and lowest under drought, but ID was not related to differences in CV^2^ or size depression. Similarly, in the hemiparasite *R. alectorolophus*, the CV^2^ of plants grown with different host species was independent of stress intensity and ID (Sandner & Matthies, 2017). In contrast, in animal studies, stress intensity, phenotypic variation, and ID were correlated (Long et al., 2013; Reed et al., 2012). Thus, differences in ID among environments are sometimes related to changes in phenotypic variation, but this does not appear to be a general pattern.

### 4.2 Effects of the stresses and inbreeding on functional traits

Plants of *A. vulneraria *strongly altered their functional traits in response to the various stress treatments. Plants allocated relatively more biomass to their roots under drought and nutrient deficiency, and allocated more biomass to above‐ground parts when subjected to defoliation, shade, and waterlogging. These responses are in line with the functional equilibrium hypothesis which posits that plants should increase the proportional growth of those plant organs that are responsible for the uptake of the most limiting resource (Brouwer, 1963; Poorter et al., 2012; Thornley, 1972). Furthermore, *A. vulneraria* modified their leaf traits in response to the different stresses. For example, plants grown under shade increased the chlorophyll content per mass, SLA, and petiole length, but decreased LDMC. These physiological responses in leaf traits are part of the shade avoidance syndrome and optimize light uptake under low‐light conditions (Franklin, 2008; Lichtenthaler et al., 1981).

In contrast to the stress treatments, inbreeding had no effect on most of the functional traits studied, except for leaf chlorophyll content which was reduced in selfed plants. Other studies have also found that leaf chlorophyll and other traits related to photosynthesis are highly susceptible to inbreeding (Kittelson et al., 2015; Norman, Sakai, Weller, & Dawson, 1995; Sandner & Matthies^2018^), which can severely affect the overall fitness of selfed individuals (Sletvold, Mousset, Hagenblad, Hansson, & Agren, 2013; Willis, 1992). Effects of inbreeding on phenotypic plasticity in functional traits may translate into environment‐dependent ID (Cheptou & Donohue, 2011). For example, plants from small, probably more inbred populations were limited in their response in leaf length to competition indicating maladaptation (Fischer, Kleunen, & Schmid, 2000). In *S. carolinense*, inbred individuals were more susceptible to herbivores, since they produced less phytohormones after leaf damage, which, in turn, limited compensatory leaf growth and carbon storage in roots (Campbell et al., 2014; Campbell, Thaler, & Kessler, 2013), and in *Echinacea angustifolia*, lower tolerance of aphid herbivory of inbred plants exacerbated ID (Shaw, Wagenius, & Geyer, 2015). In *A. vulneraria,* however, inbreeding did not affect phenotypic plasticity, as the pollination × stress interaction was not significant for any of the studied traits. This may explain why we found no differences in ID in biomass among environments. Similarly, different levels of inbreeding and six stress treatments did not consistently affect 12 functional traits in *Phlox drummondii* (Schlichting & Levin, 1986), and inbreeding hardly influenced any plant traits of *Mimulus ringens *exposed to different levels of moisture (O'Halloran & Carr, 2010). This suggests that in spite of ID in growth, most stress responses may be quite robust to inbreeding.

### 4.3 Effects of the stresses and inbreeding on the nodulation of the legume

Legumes like *A. vulneraria* are capable of forming nodules which host rhizobia that transform atmospheric N_2_ into ammonium and thus provide the plants with nitrogen and in exchange are supplied with assimilated carbon. This interaction is most beneficial for both partners under high light but low nutrient conditions (Lau et al., 2012), which explains the observed increase in the number of nodules under nutrient deficiency in *A. vulneraria*. In contrast, waterlogging leads to hypoxia in the root system, and although the rhizobia of some legumes can resist flooding by morphological adjustments (Minchin & Summerfield, 1976; Thomas, Guerreiro, & Sodek, 2005), this stress was detrimental for nodule formation in *A. vulneraria*. Plants also formed fewer nodules under drought, shade, and defoliation, which may have been due to either direct adverse effects on the bacteria or a reduced supply of assimilated carbon to the bacteria (Lau et al., 2012; Vicente, Pérez‐Fernández, Pereira, & Tavares‐de‐Sousa, 2012; Zahran, 1999).

Selfed individuals of *A. vulneraria *had fewer and smaller nodules than open‐pollinated individuals. Moreover, ID in nodule traits was stronger than ID in fitness traits indicating that the mutualism is particularly sensitive to inbreeding. Such negative effects of inbreeding on nodule formation may contribute to ID in fitness, as has been shown for other mutualistic interactions between plants and soil microbiota (Botham, Collin, & Ashman, 2009). However, nodule density (number of nodules per root mass) of *A. vulneraria *was not affected by inbreeding, which indicates that the effect of inbreeding on the formation of nodules was related to its effect on plant size.

## 5 CONCLUSIONS

We found significant negative effects of inbreeding and severe effects of abiotic stress on growth, leaf chlorophyll content, and root nodulation in the studied *A. vulneraria* population. However, the effect of inbreeding on the various plant traits was not influenced by stress type or by stress level and was independent of the novelty of the stresses to the plants. Although we studied only plants from one alpine population, our results are in line with those of other studies and suggest that there is no general pattern of the effects of abiotic stresses on ID and in particular no general increase in ID with stress intensity.

## CONFLICT OF INTEREST

None declared.

## AUTHORS’ CONTRIBUTIONS

FR, TMS, and DM conceived and designed the experiments. TMS conducted a pilot study and collected the original seeds. FR performed the experiments. FR, TMS, and DM analyzed the data and wrote the manuscript.

## Data Availability

Data from this study are available from the Dryad Digital Repository: https://doi.org/10.5061/dryad.3vq00p7 (Rehling, Matthies, & Sandner, 2018).
