# Supplementary material for: Responses of a legume to inbreeding and the intensity of novel and familiar stresses
Source: Ecol Evol. 2019 Jan 15;9(3):1255–67. doi: 10.1002/ece3.4831 (PMC6374648; doi:10.1002/ece3.4831)
Supplement: Supplementary file 1 [file ECE3-9-1255-s001.docx]

**Supporting information – Appendix 1**

Finn Rehling, Diethart Matthies and Tobias M. Sandner (2018):

**Responses of a legume to inbreeding and the intensity of novel and familiar stresses**

| **Table S1**: Analyses of covariance of the effects of biomass, open vs. self-pollination, stress type and stress level on fitness-related traits (TLA, log-total leaf area; and NL, number of leaves), biomass allocation (LMF, leaf mass fraction; SMF, stem mass fraction; and RMF, root mass fraction) and other functional plant traits (SLA, log-specific leaf area; LDMC, leaf dry matter content; Petiole, log-petiole length; and Chl, leaf chlorophyll content) of *A. vulneraria*. *, p < 0.05; **, p < 0.01; ***, p < 0.001. | | | | | | | | | | | | | | | | | | | |
| --- | --- | --- | --- | --- | --- | --- | --- | --- | --- | --- | --- | --- | --- | --- | --- | --- | --- | --- | --- |
|  |  | Fitness traits | | |  | Functional plant traits | | | | | | | | | | | | |  |
|  |  | TLA |  | NL |  | LMF |  | SMF |  | RMF |  | LDMC |  | SLA |  | Petiole |  | Chl |  |
| Source | DF | F |  | F |  | F |  | F |  | F |  | F |  | F |  | F |  | F |  |
| Total dry mass | 1 | 7358.96 | *** | 1119.17 | *** | 208.59 | *** | 27.47 | *** | 136.93 | *** | 4.33 | * | 58.96 | *** | 198.09 | *** | 48.70 | *** |
| Stress type | 4 | 227.10 | *** | 17.55 | *** | 55.79 | *** | 42.39 | *** | 65.61 | *** | 72.47 | *** | 137.45 | *** | 57.23 | *** | 78.79 | *** |
| Stress level | 2 | 35.40 | *** | 18.97 | *** | 0.68 |  | 22.29 | *** | 4.93 | ** | 28.89 | *** | 30.28 | * | 3.40 | * | 15.86 | *** |
| Stress type * level | 8 | 84.69 | *** | 13.56 | *** | 24.45 | *** | 19.46 | *** | 26.07 | *** | 24.50 | *** | 51.04 | *** | 22.99 | *** | 27.94 | *** |
| Open vs. Self-Pollination | 1 | 0.79 |  | 0.10 |  | 0.14 |  | 5.41 | * | 1.38 |  | 1.42 |  | 0.31 |  | 2.24 |  | 6.28 | * |
| Pollination * Stress type | 4 | 0.19 |  | 0.57 |  | 0.52 |  | 0.47 |  | 0.35 |  | 0.28 |  | 0.09 |  | 1.53 |  | 0.50 |  |
| Pollination * Stress level | 2 | 0.53 |  | 2.28 |  | 0.04 |  | 0.29 |  | 0.13 |  | 0.03 |  | 0.70 |  | 0.02 |  | 0.24 |  |
| Poll. * Stress type * level | 8 | 1.73 |  | 0.48 |  | 0.43 |  | 1.77 |  | 1.03 |  | 1.22 |  | 1.14 |  | 1.62 |  | 0.69 |  |
| Residual | 262 |  |  |  |  |  |  |  |  |  |  |  |  |  |  |  |  |  |  |





**Figure S1**: The absolute response of the fraction of total biomass of *A. vulneraria* represented by leaves (LMF; blue line), stems (SMF, grey line) and roots (RMF, red line) to three levels (benign, intermediate and high) of five types of stress: (a) drought, (b) waterlogging, (c) nutrient deficiency, (d) shade, (e) defoliation. Means ± 1 SE.





**Figure S2**: The effect of three levels (benign, intermediate or high) of different types of stress (drought, waterlogging, nutrient deficiency, shade, defoliation) on (a) leaf area, (b) number of leaves, (c) specific leaf area, (d) leaf dry matter content, (e) length of the longest petiole, (f) probability of nodule formation, (g) size of the largest nodule and (h) nodule density in *A. vulneraria.* Means ± 1 SE.
